# Point Pattern Analysis (PPA) as a tool for reproducible archaeological site distribution analyses and location processes in early iron age south-west Germany

**DOI:** 10.1371/journal.pone.0297931

**Published:** 2024-03-13

**Authors:** Giacomo Bilotti, Michael Kempf, Eljas Oksanen, Lizzie Scholtus, Oliver Nakoinz

**Affiliations:** 1 Institute of Pre- and Protohistory, Christian-Albrechts-Universität zu Kiel, Kiel, Germany; 2 CRC1266 –Scales of Transformation, Project F6 ‘Population Dynamics and Socio-environmental Transformation’, Kiel University, Kiel, Germany; 3 Department of Environmental Sciences, Quaternary Geology, University of Basel, Basel, Switzerland; 4 Department of Cultures, Marie Skłodowska-Curie Fellow, University of Helsinki, Helsinki, Finland; 5 CRC1266–Scales of Transformation, Project A2 ’Integrative Modelling of Socio-Environmental Dynamics’, Kiel University, Kiel, Germany; Austrian Academy of Sciences, AUSTRIA

## Abstract

Point Pattern Analysis (PPA) has gained momentum in archaeological research, particularly in site distribution pattern recognition compared to supra-regional environmental variables. While PPA is now a statistically well-established method, most of the data necessary for the analyses are not freely accessible, complicating reproducibility and transparency. In this article, we present a fully reproducible methodical framework to PPA using an open access database of archaeological sites located in south-west Germany and open source explanatory covariates to understand site location processes and patterning. The workflow and research question are tailored to a regional case study, but the code underlying the analysis is provided as an R Markdown file and can be adjusted and manipulated to fit any archaeological database across the globe. The Early Iron Age north of the Alps and particularly in south-west Germany is marked by numerous social and cultural changes that reflect the use and inhabitation of the landscape. In this work we show that the use of quantitative methods in the study of site distribution processes is essential for a more complete understanding of archaeological and environmental dynamics. Furthermore, the use of a completely transparent and easily adaptable approach can fuel the understanding of large-scale site location preferences and catchment compositions in archaeological, geographical and ecological research.

## Introduction

Over the past years, Point Pattern Analysis (PPA) has gained significance in archaeological research [1,2]. PPA examines observations that may include diachronic samples in various environmental settings or socio-cultural variables. The computational techniques used in PPA are very flexible and can easily be scaled up to interregional or international levels or scaled down to site-specific studies [e.g. 3–6]. In addition, computational PPA has contributed to the current debate on FAIR data principles (Findable, Accessible, Interoperable, and Re-usable [7] and FOS data analysis (Free Open-Source). Archaeological research has moved towards the adoption of open data, open science policies, free data accessibility and transferrable workflows that allow analyses to be replicated, comparing different regional and chronological settings and site compositions [8–11]. In this context, we advocate computational reproducibility to enhance transparency of analysis and its results [8,12], as well as empirical reproducibility that allows the transferability of code to new data and to other domains for achieving similar results [12–14]. Our aim is also to promote interdisciplinary collaboration and the exploration of synergies across archaeology and environmental sciences–and the humanities and natural sciences in general–through shared computational methods.

In this article we apply PPA to gain insights into site distribution patterns and their relationship to environmental variables, and to provide a reproducible PPA workflow for researchers across all disciplines in archaeology. For this, the only requirement is a set of site observations with geographical coordinates (the point pattern) and environmental explanatory covariates (such as elevation captured in a digital elevation model). This workflow enables modelling and visualising large archaeological site distribution patterns, both internally with reference to interactions between the sites, and externally in relation to environmental and socio-cultural variables.

Our aim is to understand site distribution patterns as functions of underlying explanatory variables and to produce comprehensive prediction models of potential archaeological site dispersal in south-west Germany. We chose a large site database covering the Early Iron Age in southern Germany with a total sample size of n = 3625 to best fit the analytical approaches presented in this article. The sample is distributed across Baden-Württemberg and distinguishes graves and settlements during the Hallstatt and early La Tène period of Central Europe. From the data, our main goal is to identify land-use preferences and regional differences in site location patterns in an inductive model.

The analytical code, written in the R programming language [15], is accessible as an R Markdown file (.rmd) (see code availability to this article). The code and the workflow can be used to fit different site distribution patterns across the world and make it possible to compare the results of the analysis with other regional contexts, allowing researchers to draw conclusions about the large-scale evolution of archaeological sites in diverse environmental contexts.

The paper is structured as follows: first, we introduce the archaeological data with a brief description of the study region’s chronological development; second, we describe the environmental covariates; finally, we offer a detailed methodical workflow including statistical modelling techniques of point processes.

### Archaeological database

**Fig 1:**
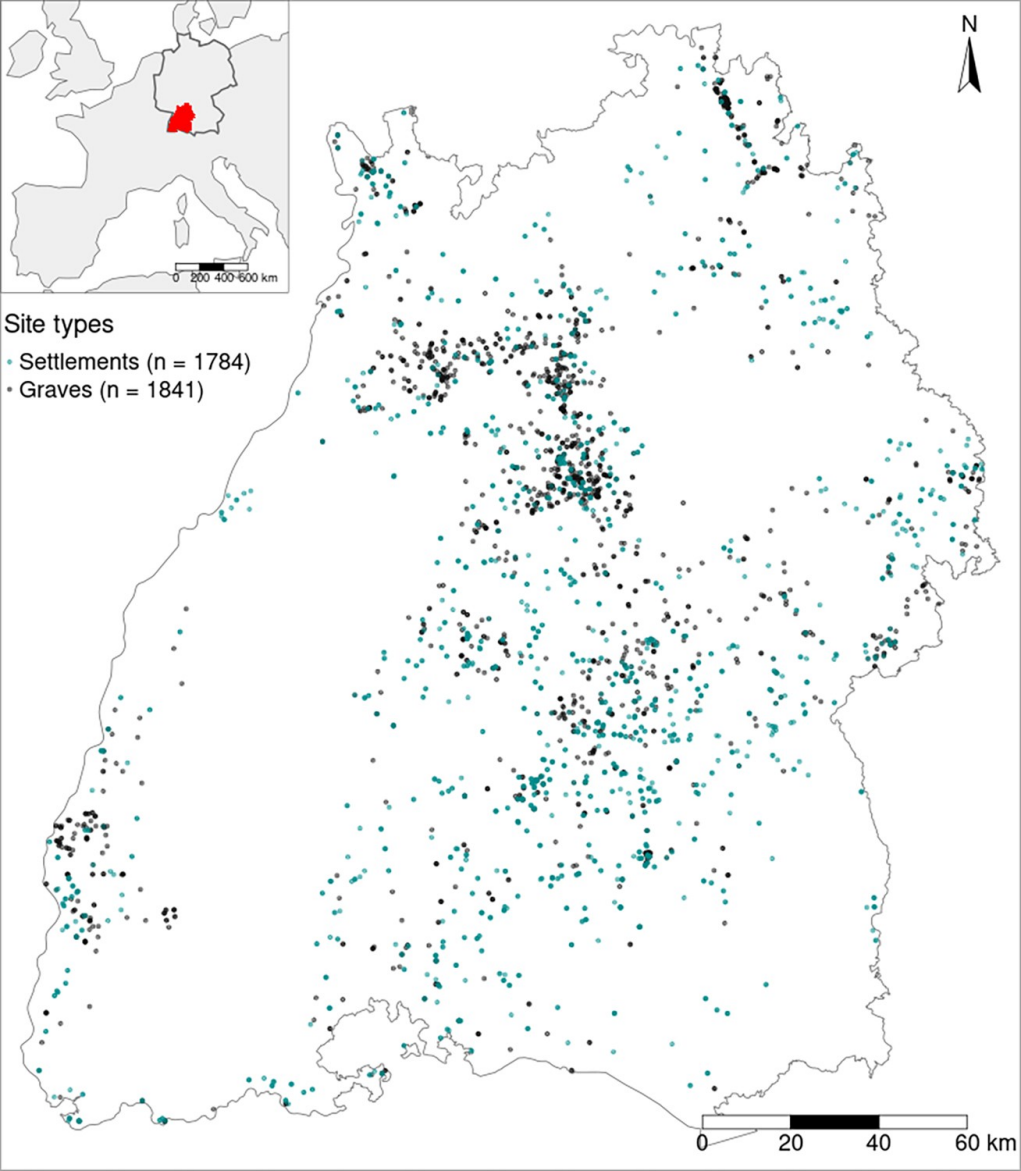
Distribution of settlements and graves from the Hallstatt and early La Tène period in the federal state of Baden-Württemberg, Germany. On the top left corner the study area is highlighted on a map of western Europe. Administrative borders made with Natural Earth. Free vector and raster map data @ naturalearthdata.com. Data is public domain.

We examined Early Iron Age archaeological sites in south-west Germany, including 1784 settlement and 1841 burial sites from the Hallstatt (Ha) and Early La Tène (LT A) periods (Fig 1), with a majority of the sites dating to the Hallstatt period. Within the Hallstatt period, phases C and D are analysed separately and combined due to the limited precision in dating of many of the sites in the database. Visual examination of the site distributions suggests a distinct concentration of settlements (of both periods) in the lower elevation regions in north-central Baden-Württemberg. Furthermore, there is a significant site intensity of Hallstatt graves in higher elevated areas in south-central Baden-Württemberg. We do not observe the same pattern among burial sites dating to the La Tène period.

The data used in this analysis derives from an inventory carried out within the project “Siedlungshierarchien und kulturelle Räume” (SHKR: [16]) and was published online in 2013. The geographical boundaries of this study cover the administrative boundaries of the state of Baden-Württemberg and the data includes the chronological range from the Hallstatt C to the La Tène A period, representing the Early Iron Age (800 to 400 BC ca.). The SHKR project aimed to explore various aspects such as territorial and social hierarchy, economy and centrality. Initially, the data collected was mainly related to settlements, but funerary information was later incorporated to provide a more balanced dataset [17]. Today, this database represents an advance in archaeological research in the region and the chronological period, although it could be argued it overrepresents funerary data compared to settlement sites–which is a common issue in archaeological settlement distribution analysis [18,19].

### Chronological development

The Early Iron Age in Europe, particularly the Hallstatt period, is marked by numerous societal, economic and cultural changes that also have repercussions on the occupation of the territory in this region [20]. During the Hallstatt C period the population was self-sufficient, composed of small scattered villages and agricultural territories distinguished by quadrangular enclosed farms and hilltop settlements [19]. In this period, the practice of building burial mounds is established, although cremation burials are still practiced in flat graves. These monuments were generally used by the same community over several decades, sometimes extending through the entire Early Iron Age [21,22].

During Hallstatt D, hilltop settlements became more widespread [23] and there is evidence for a new form of elite emerging. The so-called “princely seats or sites”, as defined by Kimmig [24], were subject to intensified research [25]. These extensive and fortified agglomerations attracted further dwellings around them. Large tumuli are found at the bottom of slopes or in the valleys with richly endowed burial chambers including imported Mediterranean goods [21]. Four of these princely seats are known in Baden-Württemberg, with the Heuneburg being the most extensively investigated [26]. Graves also provide important clues to settlement structures. Müller-Scheeßel [27], for example, was able to work out an expansion of the settlement landscape on the basis of the graves, which he does not see exclusively as monuments of the elite.

The transition to the La Tène period is widely recognised as a transformational period in this part of Europe. The princely seats were abandoned before or during the Early La Tène period and open settlements developed in the plains and valleys. A study using Geographic Information Systems analysis in an adjacent area discussed whether pastoral economy became dominant at the shift from the Hallstatt to the La Tène period [26]. In the funerary culture, we observe a development towards flat graves from the end of the La Tène A and in particular during the A2 phase. Lavishly furnished chariot graves, featuring ceremonial drinking vessels and imported artefacts are, however, still found [18,21]. In general, the region of Baden-Württemberg stands out in its density of identified sites. The larger sites were well integrated in the international trade and communication networks, and the surroundings appear to have housed a relatively high number of inhabitants, in some cases exhibiting characteristics of proto-urban centres.

The high number of larger sites with central functionalities known for this region has been in the focus of past archaeological research. Previous studies on site distribution mostly sought to define and characterise networks and centrality, focusing on these specific settlements and attempting to delimit their spheres of influence [16,28,29]. Similarly, earlier research on graves and tumuli often aimed at identifying so-called elite sites and exploring the characteristics that set them apart [26]. The past decade has also introduced varied perspectives on the nature of these “elites”, particularly in differentiating between status and prestige [30]. Although recent studies in the area have suggested that settlement locations should be considered in relation to other settlements [23,31] (referred to as ’first order point pattern properties’ in our terminology), there has been a lack of an analytical workflow that integrates both settlement and grave locations with geographical contexts. Moreover, central place theoretic concept has been applied to the distribution of archaeological sites in southern Germany [32,33], underscoring the necessity for a more comprehensive and interdisciplinary approach.

### Covariate description

There are multiple environmental covariates that can be used to estimate site distribution patterns, including approaches from studies of ecological niches [e.g., 34] and archaeological modelling [e.g., 9,35,36]. Here, we focus on five explanatory covariates (described below) that are tested for site preferences across the different chronological periods (Hallstatt and La Tène), and for the two different types of archaeological record (settlements and graves). The environmental parameters contribute, together with archaeological explanatory covariates, to a predictive model of potential site distributions in the study area.

The five selected environmental covariates are elevation, slope, topographic wetness, topographic position, and distance to running fresh water. A TanDEM-X digital elevation model with a 90 m resolution is used as a basis to calculate the slope, the Topographic Wetness Index (TWI) [37] and the Topographic Position Index (TPI) [38] of the study area. The distance raster is calculated using simple map algebra using as input the EU-Hydro River Network Database. Only the rivers with a Strahler order above 4 were considered. We use the R packages *Rsagacmd* [39], *sf* [40], *terra* [41] and *whitebox* [42] for this purpose. The script used to calculate the covariates is the *01_Geom_Parameters*.*R* paper in the analyses folder available in the *GitLab* repository (see code availability section). Numerous countries or institutions offer higher-resolution DEMs, sometimes freely accessible. There are, however, areas in the world where this is not the case. To ensure reproducibility, we made the decision to utilise a 90 m resolution DEM, which matches the resolution of the globally available SRTM data.

Slope measures the steepness of a surface and is essential in understanding water flow, sedimentation, and energy expenditure [43]. The TWI was developed to assess soil moisture based on land topography and it has an important impact for plant growth and hydrology [37]. The TPI is a quantitative measure used in environmental analysis to describe the topographic position of a point on a landscape relative to its surroundings. Specifically, positive TPI values indicate ridges or hilltops, negative values indicate depressions or valleys whereas values around 0 indicate flat areas or mid-slope values [38].

All the analyses presented here have been carried out using the WGS 84 / UTM zone 32N reference system (EPSG: 32632). It is a Universal Transverse Mercator coordinate system based on the World Geodetic System 1984 datum. If the raw data was in a different coordinate reference system, it has been reprojected. The scripts required for this reprojection are available in the GitLab repository.

## Methods and data

**Fig 2:**
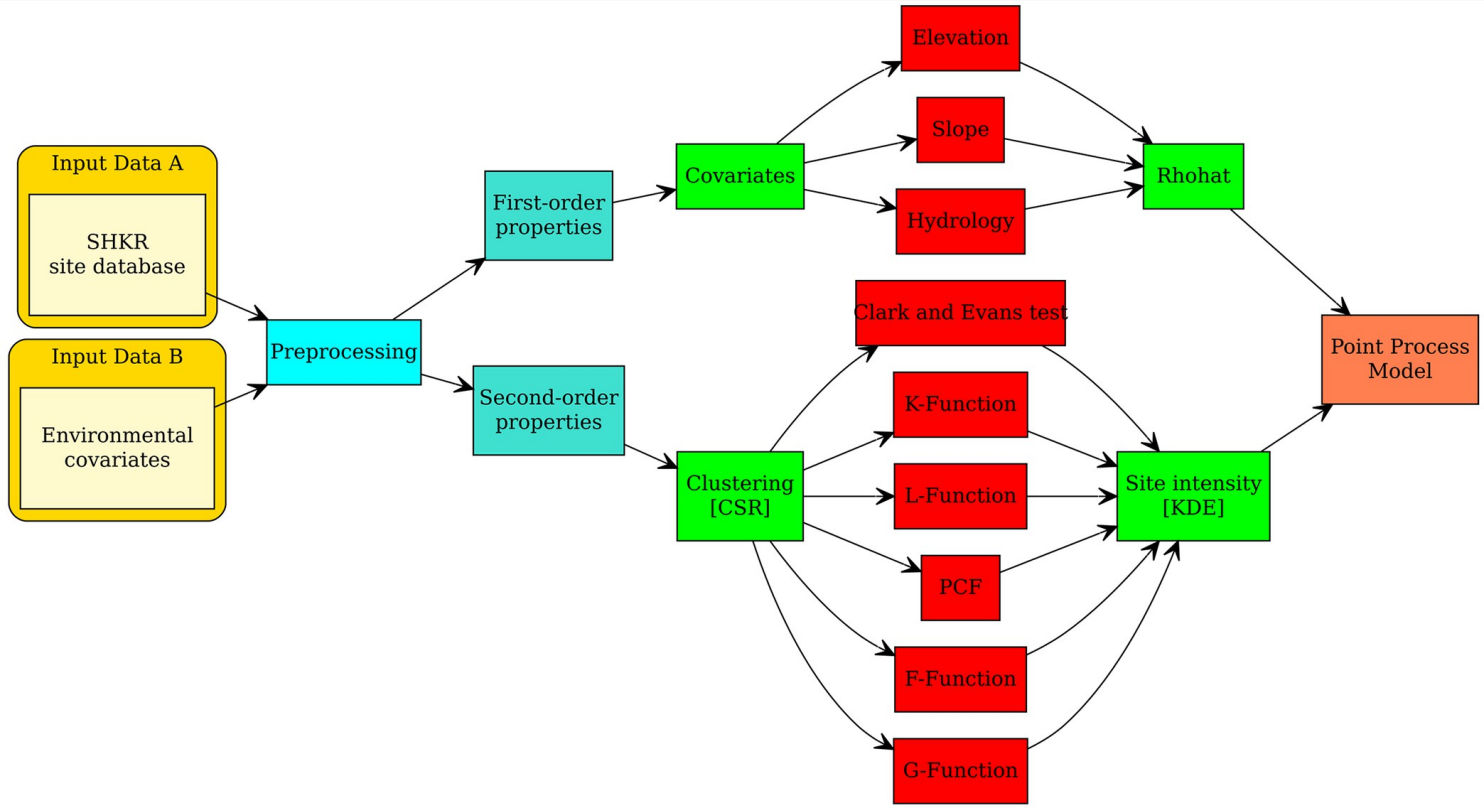
Methodical workflow of the Point Pattern Analysis (PPA) of this paper. We evaluate the spatial behaviour of point data (input data A) compared to environmental data (input data B) in a reproducible workflow using R software.

PPA is based on two principles, commonly referred to as first-order and second-order properties [44,45]. First-order properties refer to the intensity of a process in relation to an external covariate, such as the number of sites in a particular area and an environmental explanatory variable. The location of a point is affected by the underlying area’s structure, but not by the location of other points [46]. On the other hand, second-order properties describe inherent spatial dependency within the point pattern [47], such as attraction or repulsion, and occur when a point’s location is influenced by the presence or absence of other points [48]. Ecological studies commonly use point pattern analyses [48,49], and these methods have increasingly been applied in archaeological research [2,6,35,36,50,51]. Point pattern analysis provides a clear terminological frame that naturally highlights issues of interaction with the environment (first order properties) and with other sites (second order properties) (see Fig 2). In particular the latter is of greatest relevance for social interpretations. A straightforward interpretation of the assumed interactions revealed by the point pattern, however, is usually difficult due to the presence of complex first order effects. The comparison of different time slices circumvents this limitation to a certain degree, and allows the analyst to infer and quantify changes in site interactions between the time slices. This transparent workflow makes the basis of the approach (as well as its limitations) visible, and being universally adoptable improves the research reproducibility. The high abstraction level of formal point pattern analysis ensures the comparability of the results from different regions, even if the data and the site categories are different. Furthermore, the generalised approach of point pattern analysis is not restricted to specific disciplines, which simplifies interdisciplinary understanding and hence promotes interdisciplinary communication and collaboration.

### Spatial interactions

PPA powerfully assists in interpreting large point patterns that would otherwise be difficult or impossible to investigate simply by visually examining a distribution map. To illustrate our approach we will first carry out a sequence of second-order analyses to explore structural properties in the form of spatial interactions within several point patterns that represent sites of different types and chronological periods. A starting point in applying PPA is to detect what spatial trends a spatial point pattern follows. We divide point patterns into three idealised categories: clustered patterns where points congregate into one or more clusters across a study area, regular (or dispersed) patterns where individual points repel each other, or patterns that are spatially random [45].

Spatial randomness (CSR—complete spatial randomness, formally described as a homogeneous spatial Poisson process) is indicative of the pattern’s character, and suggests that the individual observations occurred independently of each other and without detectable first- or second-order properties [3]. CSR assumes that an event (point) in one location does not affect events in other locations.

A well-established PPA method for analysing the spatial properties of a point pattern is the Clarks and Evans test (also known as the nearest neighbour test). The Clarks and Evans test was originally developed in ecological studies and applied, for example, to study the plant species distributions [52]. The test produces a single value (r), where r>1 indicates clustering, r<1 dispersal and r = 1 Complete Spatial Randomness as the governing tendency across the whole point pattern. Our results (at region-wide scale across Baden-Württemberg) do not show any chronological variation, with values more dependent on the typology of sites. In general, settlements tend to have values between 0.41–0.46, whereas graves have a higher clustering rate between 0.51–0.60. The Clark and Evans test, however, does not account for point processes taking place at different spatial scales of a point pattern [53]. In the above example, a series of neighbouring settlements located along a river valley might have a dispersed pattern owing to competition for agricultural land when examined at the range of a few kilometres. But as we zoom out to a scale of dozens or a hundred kilometres, we can see that settlements cluster along the river and its tributaries. Therefore, these results should be interpreted with care and only as a first step before performing more robust tests.

The K-function (also known as Ripley’s K) is among the most well-known methods capable of investigating point processes at multiple scales [53]. The number of neighbours for each point is cumulatively calculated in concentric circle intervals, obtaining a frequency distribution of average point intensity at intervals of r. Plotted on a graph against a simulated Poisson distribution representing CSR, the results can be read in a manner similar to the Clark and Evans’ test: if at a given scale point the observed point distribution (a black line) is below the theoretical distribution the pattern is dispersed, and if above it is clustered. The specificity of the analysis can be enhanced by conducting n number (here n = 999) Monte Carlo simulations [54,55] of random point distributions in the study region and visualising the combined results as a 95 percent (i.e. within two standard deviations) confidence interval (referred to as envelope); where the function falls within this confidence envelope the pattern can be considered as spatially random [1,4,56].

The L-function derives from the K-function and is simply a mathematical straightening of the Poisson line to improve the visualisation of the results. The Pair Correlation Function (PCF) is similarly calculated, but instead of cumulatively adding neighbouring points together the numbers are counted within concentric doughnut-shaped neighbours; this has the advantage of picking up more fine-grained structural details on clustering or dispersal at different scales [3]. The F-function and the G-function are less known but (as we will demonstrate) can be very useful in archaeological site distribution analyses as complementary functions [4]. The functions therefore evaluate whether the spatial characteristics of archaeological observations differ from a theoretical distribution based on CSR, and offer a powerful interpretative tool for examining the processes that gave rise to these point patterns. The G-function, referred to as the "nearest-neighbour distance distribution function," offers a comprehensive synthesis of the cumulative distribution associated with the nearest neighbour function. Differing from the aforementioned nearest-neighbour distance, this function effectively retains information pertaining to the spatial configuration of the point process. The G(r) value represents the likelihood of encountering a neighbouring point at a distance r from any point X. Correspondingly, the F function, also known as the "empty space function," describes the empty-space attributes of a point process. The F(r) value indicates the probability of a point’s presence at a distance r from a location X. Both functions exhibit cumulative and stationary characteristics [4,57].

### Spatial intensity

The spatial distribution can be examined through a kernel density estimation (KDE), a data smoothing technique that measures the expected number of points per unit area as intensity of the point process and thereby summarises distribution density [57]. During analysis, a kernel shape is moved across the study area. At each location the intensity of observations (in this case, point locations representing settlements or graves) falling under the shape is calculated using a quadratic equation, and the value mapped at the centre of the shape’s current location. The results are expressed as a continuous surface [3,58–60]. The granularity of KDE is set by the bandwidth (radius of the kernel or sigma), as well as its shape. The function density in the spatstat package uses a weighted distance kernel, where points nearer its centre contribute more to the value [57,61]. A larger bandwidth results in a smoother density surface that is useful for visualising and identifying larger scale patterns, but may obscure smaller details.

Testing different bandwidths to evaluate the results may form a part of a data exploration workflow. Another approach is to perform an inductive test in which the bandwidth is derived directly from the characteristics of the data. In this study, we computationally determine the ideal smoothing sigma of the kernel for each individual point pattern using the *bw*.*diggle* function of the *spatstat* package, based on [62]. The result of the function provides a hint about the general spatial distribution of our sites, since we can roughly assume that the larger the output of the function the more distant the sites tend to be located from each other. Another advantage of the function, important given our overall goal of demonstrating an exploratory case study that can be easily reproduced in other contexts, is that it can be applied without modification to any other point pattern. Here, the optimal bandwidths calculated for settlements and graves are averaged in order to have a common sigma for all settlements and another for all graves. The *spatstat* package offers other methods for determining the optimal bandwidth. It’s noteworthy that different functions can yield notable discrepancies in results [57]. The *bw*.*diggle* function assumes an underlying Cox process, whereas the *bw*.*ppl* assumes a Poisson process. In our study, we selected *bw*.*diggle* due to its tendency to consider points as more clustered, typically resulting in a lower sigma. This choice helps prevent excessive smoothing in the final KDE. The risk of very low sigma is mitigated by averaging the outcomes across the different sub-datasets.

### First order properties

First-order properties are estimates of point patterns in relation to explanatory covariates, usually environmental parameters. Inherent in such an approach are the assumptions that particular environmental features in the landscape are more attractive than others, and that there are environmental parameters that control human action within particular distance around settlement sites. While potentially deterministic, the advantage of this approach is that it can be applied to analyse large archaeological site databases to produce highly informative estimates of preference or avoidance of particular landscape features at specific chronological periods, and further allows to trace differences between groups, chronological time-slices, or geographical areas [35,36,57,63].

#### The rhohat function and focal statistics

To calculate site intensity against environmental covariates we use the rhohat function from the *spatstat* package [64]. This method visualises attraction or repulsion of an environmental parameter, such as elevation, on the point distribution of a given subset with a user defined confidence level (in our case 95%). We can see, for example, that both Hallstatt and La Tène settlements concentrate on the lower elevations (> 500 m asl), but that there is an intriguing relative concentration of Hallstatt burial sites around ca. 800–900 m asl that is not replicated in the La Tène data. Furthermore, the approach presented in this paper makes it possible to track site location parameters not only as a spatially static component in human decision-making, but integrates a catchment composition evaluation to the analysis. Using continuous data, such as from slope gradients generated using the DEM, preferences for particular slope ranges and thus topographic roughness can be estimated.

In addition, a focal approach can be applied that aims at testing the composition of particular environmental conditions within a predefined complementary region. This has the advantage of taking into account not only the environmental condition at the exact site (represented in the data as a 2-dimensional coordinate point and therefore lacking a representative spatial extent), but also the variation of these conditions within the surrounding catchment [4,35,51]. In essence, we assign each cell the sum of the values of the surrounding cells. Calculations and analyses are conducted using the R environment and the packages *dplyr* [65], *maptools* [66], *sf* [40], *spatstat* [57] and stars [67]. Focal statistics were calculated, in which the values of the neighbouring cells were included into the calculation of each cell in a moving window. Here, we defined a radius of 5000 m for the single soil and the hydrologic Strahler data, and a radius of 1000 m for slope and elevation. The univariate density analysis produced by the rhohat function serves as a powerful initial data exploration step, with further insights afforded by the Point Process Model (PPM).

#### Point Process Models (PPM)

In order to understand our point process model we used a Poisson model fitted to our original data and interpreted the results. Such models are described by their intensity function and are fairly easy to build: given the points and the covariates the model is an equation representing this intensity–hence the expected number of points at a given radius around each point [57]. A necessary premise for applying this method is that observations are distributed following a homogeneous or inhomogeneous Poisson process. Because of the nature of the intensity function, it is generally adequate to treat point distributions as inhomogeneous Poisson processes [57].

To fit a Poisson point process model using the ppm function from the spatstat package [61], a formula needs to be defined. This formula includes the points to be modelled as well as the covariates. Multiple covariates can be used simultaneously and combined in various ways. In this study, the covariates were summed together with equal weights assigned to each covariate.

The model is fitted for each subset of points (based on period site typology). Then, in order to optimise the model, a stepwise model selection approach using the Akaike Information Criterion (AIC) is applied. The objective behind is to minimise redundancy in the model without losing predictive power [68]. This can be done using the step function from the *stats* package [15], which is a simplification of the stepAIC function from the MASS package [68].

Lastly, to examine the correlation among the various covariates and avoid overparameterization, we employed the vif function from the *car* package [69]. The function calculates the variance inflation for linear and other regression models. It is also possible to calculate correlation between the different variables before the PPM model, performing a Pearson’s correlation test using the cor function available in the *stats* package [15]. Values close to 1 indicate a lack of correlation, while values approaching 5 suggest moderate correlation. When values surpass 5, this signifies a high level of correlation. We opted for the former method because it is faster and does not require previous data preparation. The results consistently demonstrate a lack of correlation and are therefore not further discussed here (but are available in the supplementary material). This aligns with our expectations following the application of the stepAIC function, which is designed to minimise noise and simplify the model, thereby eliminating redundancy, including correlated variables.

### Potential bias detection using rbias

The modern land use and surface alterations significantly affect the formation of our known archaeological record. This implies that the spatial distribution of archaeological features is more a product of landscape development and contemporary activities, rather than being a representative sample of past events [51,70–72]. Archaeological databases in particular are susceptible to multiple biases due to the current state of knowledge, research interests, technical capacity and database formats. Thus, we employ the recently developed R-package *rbias* [73] to analyse, visualise, and interpret the impact of modern built-up and infrastructural development on the distribution of archaeological sites.

The package enables users to download *OpenStreetMap* data using the *Geofabrik* service. Here, we incorporate the features falling within the study region that consist of significant land development features, namely buildings, roads and railways. To mitigate the sharp boundaries of these features, we employ the R package *FuzzyLandscapes* [74], which uses fuzzification techniques [75]. Fuzzification allows the definition of varying ranges of impact of modern infrastructure on archaeological sites and rescale the entire surface. Here, we tested two different ranges: 250 and 1000 m. Points falling within the infrastructure are assigned a membership degree of 1 (strongest bias). As the distance from the object increases, the bias or membership degree gradually diminishes until it reaches 0 at the maximum range or beyond (in our case 250 or 1000 m).

## Results and discussion

The following section presents the PPA results from the regional case study in Germany and provides the basis for interpretation of the analytical outputs. To integrate the regional archaeological knowledge into the discussion of the methods and the results, we show what PPA and quantitative statistics in general can contribute to identifying patterns and integrating individual sites into larger explanatory frameworks. We emphasise the collaborative interdisciplinary research design of archaeology, environmental sciences, and computational approaches in the humanities, and the sciences in general, to generate knowledge beyond unilateral research approaches.

### CSR tests and spatial point processes

The point patterns have been tested for CSR using a plethora of statistical tests and functions. Detecting the spatial interaction of the events within the point pattern allows us to understand if the sample follows a random distribution.

#### Spatial interaction

**Fig 3:**
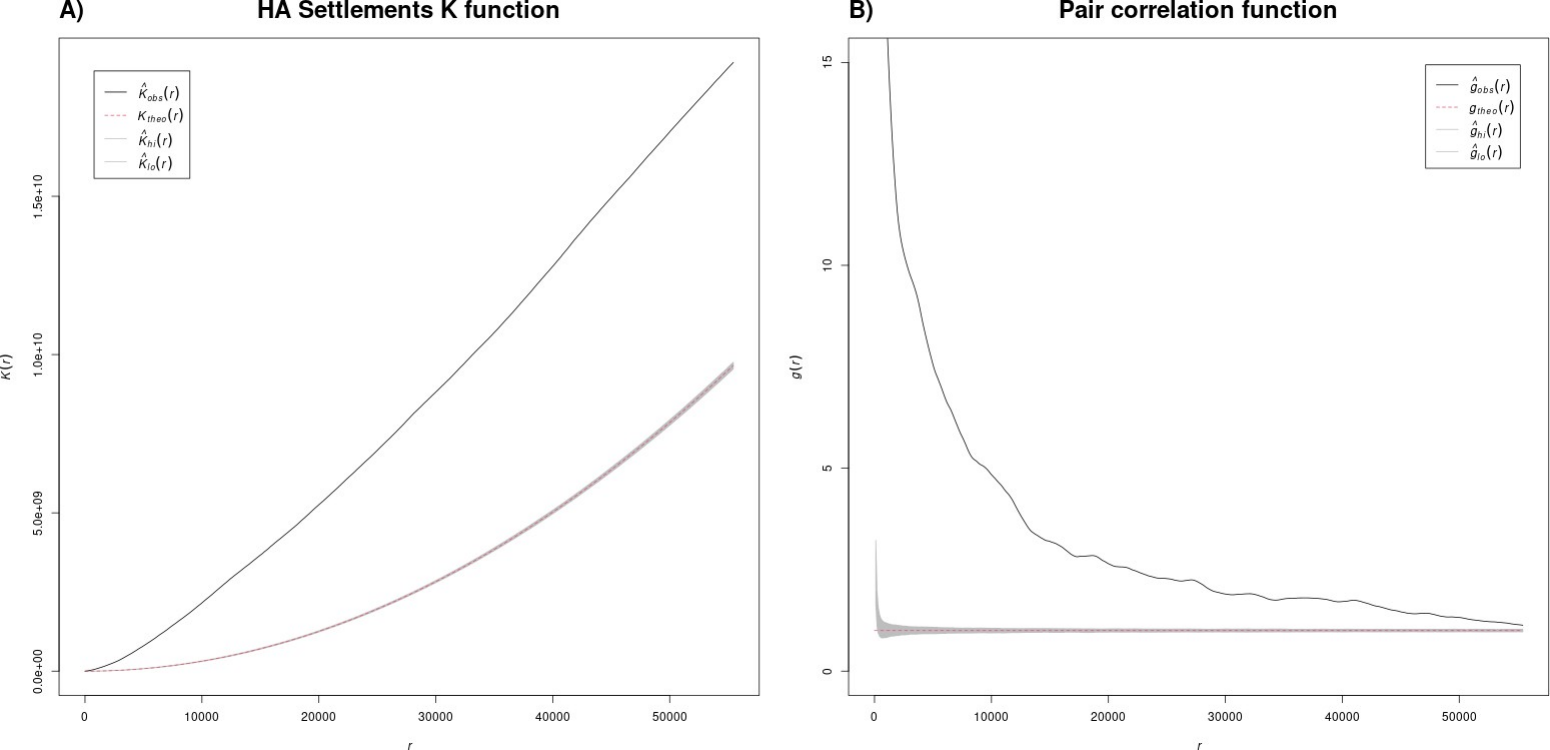
Ripley’s K and pair correlation function for Hallstatt settlements. To see all the K, L and PC functions see the GitLab repository.

Settlements from all the chronological classes considered here show spatial clustering at all distances in the Ripley’s K function and the L function (Fig 3). However, this pattern becomes less consistent in the pair correlation function, with a pattern closer or within the confidence interval for increasing radii. Similarly, graves show a clustering pattern at all scales in the K, L and pair correlation function. The only partial exception are Hallstatt C graves, which have a random distribution at increasing distances.

The results show that sites tend to be located next to each other and that at larger distances they are more likely to be found in groups, which may reflect socio-cultural decision making or more simply the reflection of environmental factors or recovery bias. To better disentangle cultural and environmental settlement choice we turn to the point process models.

**Fig 4:**
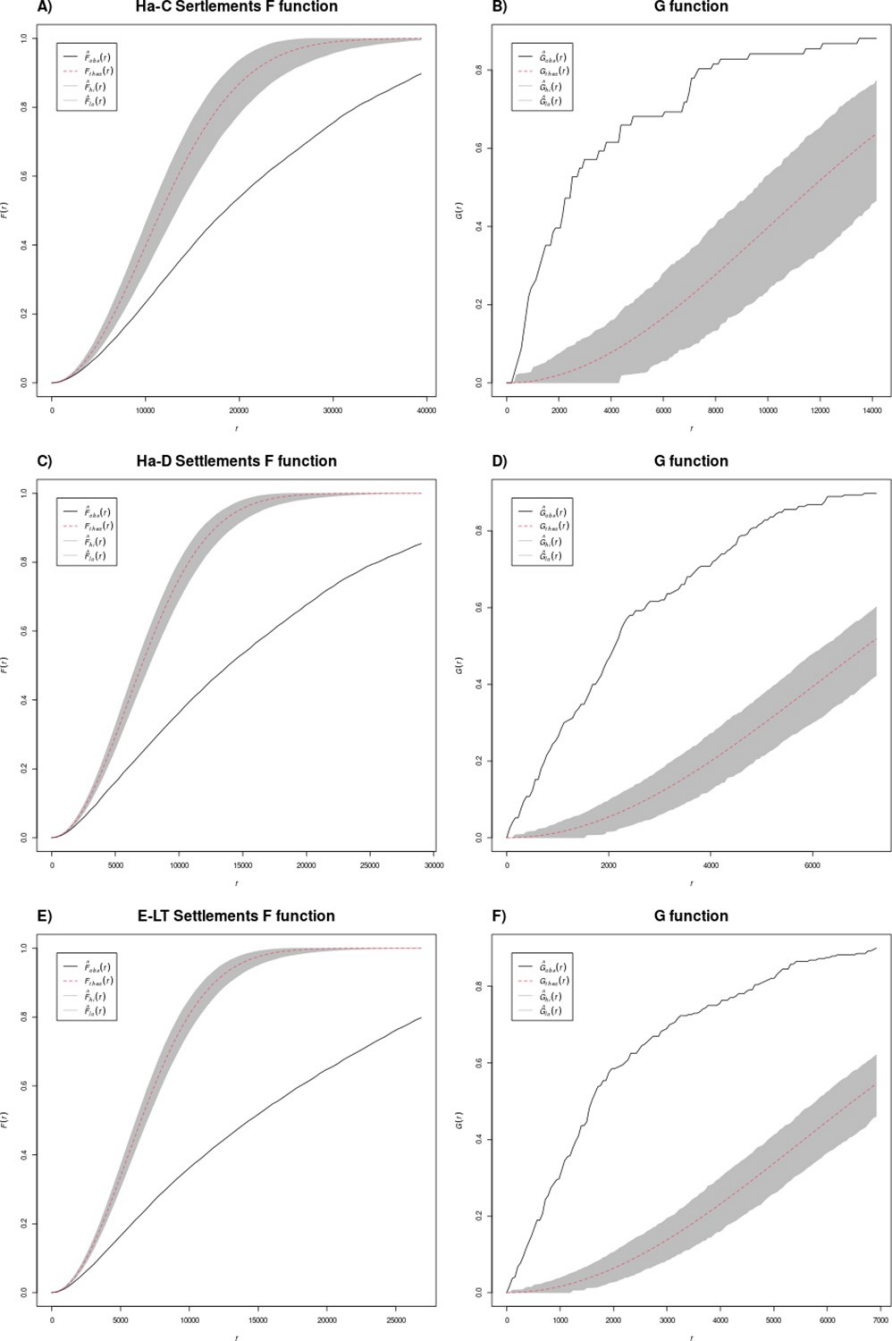
G and F function results for settlements.

**Fig 5:**
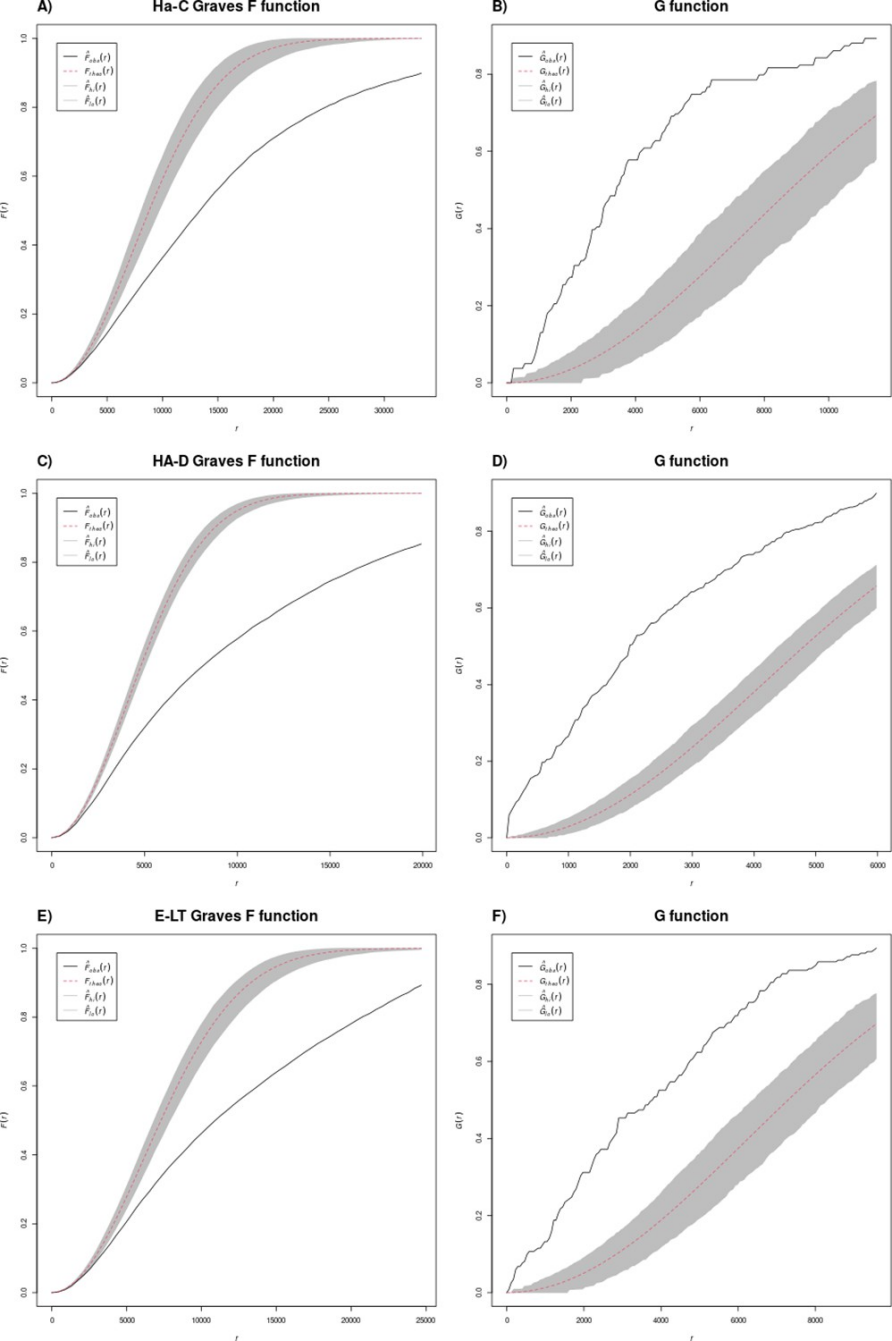
G and F function results for graves.

Since first-order effects lead to clustering in most archaeological data, it is no surprise to observe a clear clustering in all partial data sets (Figs 4 and 5). It is possible to examine the structural characteristics of the different subperiod patterns further using the G- and F-functions. In the case of the G-function, clustering can be detected from the fact that the empirical values are above the theoretical for a random distribution. This is caused by the fact that there are more short nearest neighbour distances in a clustered pattern than in a random distribution. On the other hand, in the F-function the empirical curve is below the theoretical one because it measures the distance *from* the random points *to* the clustered data points, thus the distance is greater than it would be in a random distribution. Thus, the aim here is not simply to recognise a clustered or another type of point pattern, but to distinguish the intensities and details of these patterns by evaluating the results of the different methods. In this context, the G-function proves to be particularly valuable, as the F-function results, with their “external” perspective, exhibit similar characteristics due to minimal changes in clusters across different phases in the overall picture.

The combined analysis reveals that settlements exhibit a more pronounced clustering compared to graves. This may be due to the fact that the settlements have a shorter chronological duration and their displacement can lead to multiple records in a relatively short distance. It could also be related to pull factors of the “princely seats” and their central function in the territorial organisation of the landscape [28,29]. Nevertheless, it is noteworthy that in both categories, settlements and burials, the clustering is stronger in Ha C than in Ha D and LT A, that is before the spread of hilltop and so-called elite settlements. For the settlements in Ha C, two steep areas of the curve can also be observed, indicating that dense and less dense clusters can be distinguished (Fig 4A and 4B). In the two later phases, the settlements within the 1000 to 2000 m range show a comparatively steep rise and then a bend into an approximate parallel to the theoretical curve (Fig 4C–4F). This shows that the clusters do not have a hard core, i.e. a minimum distance between the settlements, but a more or less equal distribution of nearest neighbour distances. This is not an equal distribution of points, but rather an equal distribution of all density values. Beyond the clusters, points are scattered in a relatively random manner, connecting the clearly delimited clusters with each other.

**Fig 6:**
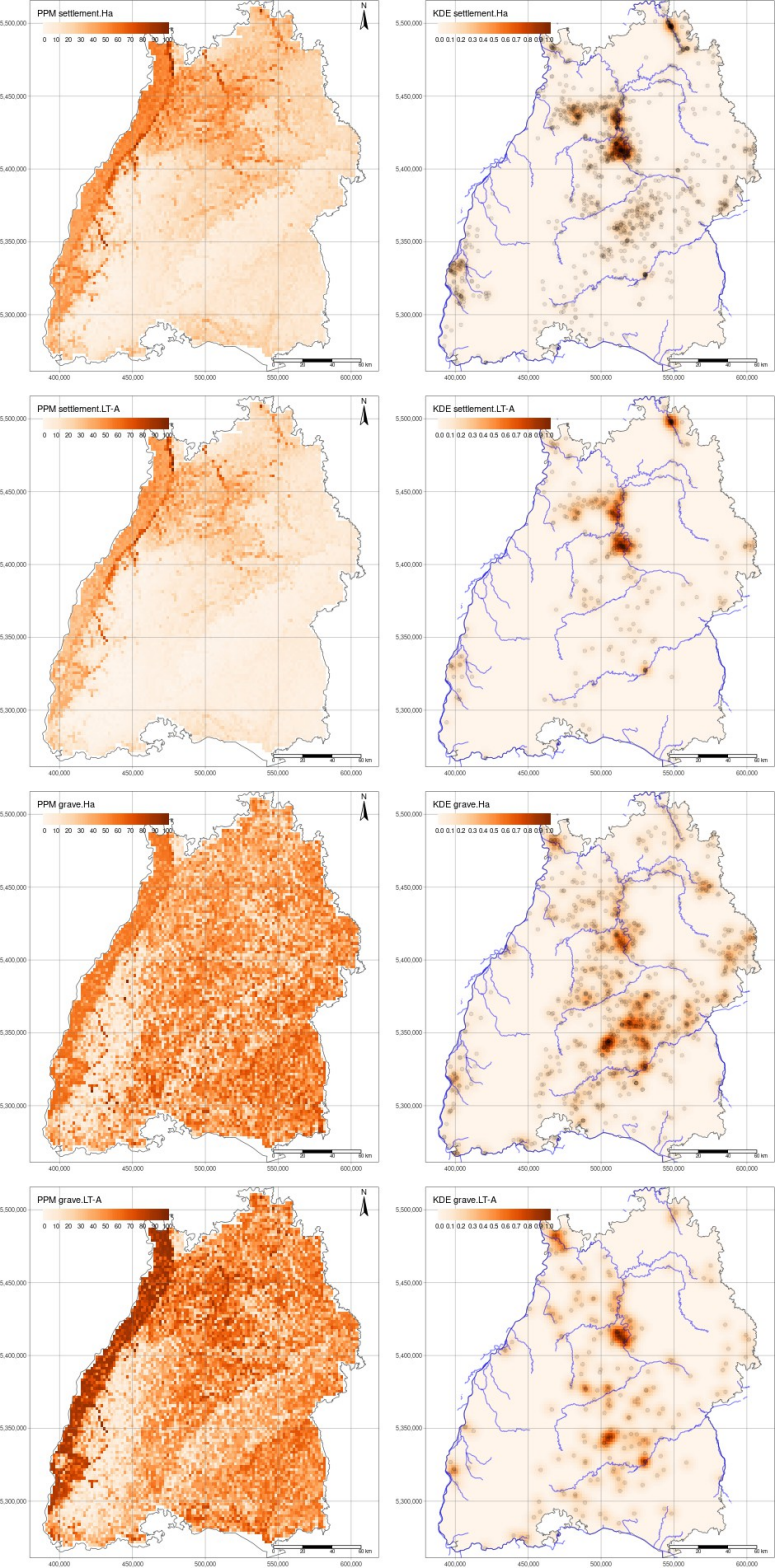
PPM (left) and KDE (right) results for selected periods. Rivers and sites are plotted with the KDE. River data generated using the full, open and free access data from the European Union’s Copernicus Land Monitoring Service information (https://doi.org/10.2909/393359a7-7ebd-4a52-80ac-1a18d5f3db9c).

This demarcation of clusters is not observed among the graves, but we observe differences between minimum site distances between the different subperiods for both site types (Figs 5 and 6, right). In Ha C a short initial minimum distance of perhaps a hundred metres can be observed (Fig 5A and 5B). In contrast, the G-function curve in Ha D shows an uptick immediately at minimum distances that is especially pronounced for cemeteries, and indicates that these cemeteries have neighbours at minimum distances, i.e. they are probably not clearly delimited spatially (Fig 5C and 5D). This may depend on how different burials or tumuli belonging to the same necropolis or spatially related may not be clearly demarcated in the database; such perspectives are important for detecting and interpreting possible recording biases in archaeological databases, which can have a substantial impact on data re-use. In Early La Tène, the curve does not show a step or offset, indicating clustering that is moderate but clear (Fig 5E and 5F).

It is important to remember that the data combine diverse regions, resulting in the partial averaging of regional differences. Nevertheless, it can be seen that the clustering is strongest in Ha C. This effect seems to counteract the concentration of power. However, it should be noted that settlement patterns do not necessarily reflect power structures, as the dominant factors at play are often related to settlement and consolidation processes

#### Intensity of the Point Process (KDE)

The point process intensity is the measure of the density of the underlying point pattern. We examined the KDE with a bandwidth of 3187 m which is the average of the optimal bandwidths obtained with the *bw*.*diggle* function in *spatstat* (Fig 6, right). For the Hallstatt settlements, we observe a strong clustering in the north-central part of Baden-Württemberg with smaller clusters towards the northern and the south-western part of the region. It is only among Ha C settlements that we observe a more balanced pattern (see also the gitlab repository for supplementary material to this article), with several clusters isolated and fairly similar in intensity, although the bulk of the site density is still located in the central and northern parts of Baden-Württemberg. Secondary but lower intensity clusters are abundant in the Swabian part of Baden-Württemberg towards the east. In Ha D the situation appears similar to the total Hallstatt settlement site dispersal. This is mostly due to the fact that Ha D settlements contribute to the majority of all Hallstatt settlements. Clusters remain in the central part of the study area, however, we observe secondary clusters towards the south with a particular high site intensity at the Heuneburg (the south-eastern cluster). During the Early La Tène period the situation seems to be continuous, with the major site intensity still located in the central and northern part of the region, building on the current archaeological interpretation of settlement and population continuity in south-western Germany [21,22,28].

Hallstatt graves have a rather different intensity compared to the settlements, with more prominent clusters located along the south-central and eastern parts of the region (Fig 6, right). Most likely, we visualise here a research bias inherent in the data, which derives from a long-standing focus on burial places compared to settlements [19]. Interestingly, this is the area located between the rivers Neckar and Danube, possibly indicating an intentionality of dominating (visually or territorially) these two major communication corridors in the area.

Another area with high grave density (but lower than the aforementioned) is located in the north-central part of the area, where also the large settlement clusters are located. This pattern is repeated with only minor differences when graves from Ha C and Ha D are considered separately. However, in the Ha D period the intensity in the central and northern parts of the region (along the river Neckar) seems higher. A possible explanation is again the difference in the number of sites, which is much higher in Ha D compared to Ha C, pointing towards a sample-size related intensity. The situation seems to slightly vary during the following Early La Tène period, with the highest intensity located in the north-central part of the region, corresponding to settlement location. However, the high-intensity areas located in the southern and south-eastern parts of Baden-Württemberg are maintained.

#### Rhohat and PPM results

**Fig 7:**
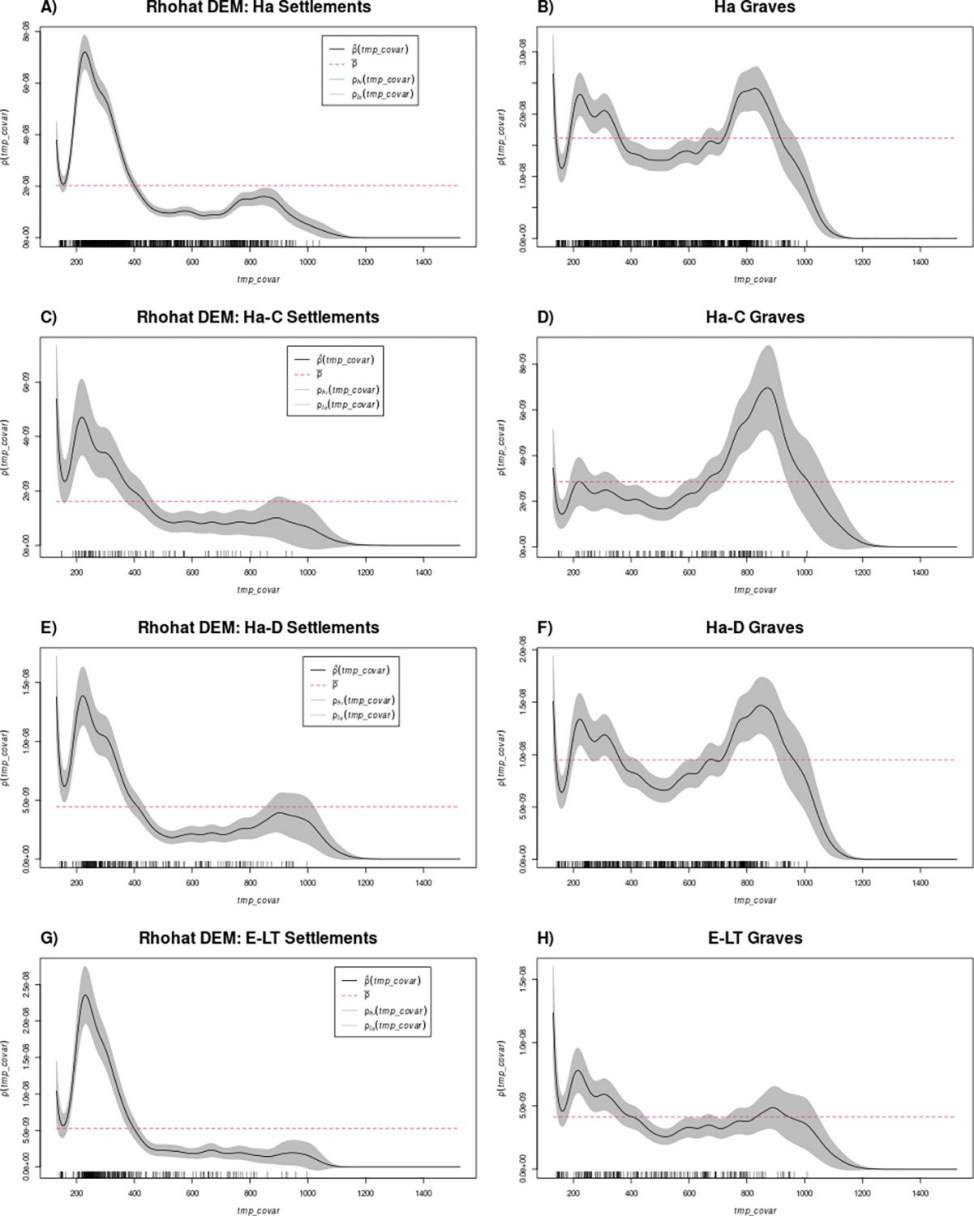
Rhohat results for settlements (left) and graves (right) vs elevation.

**Fig 8:**
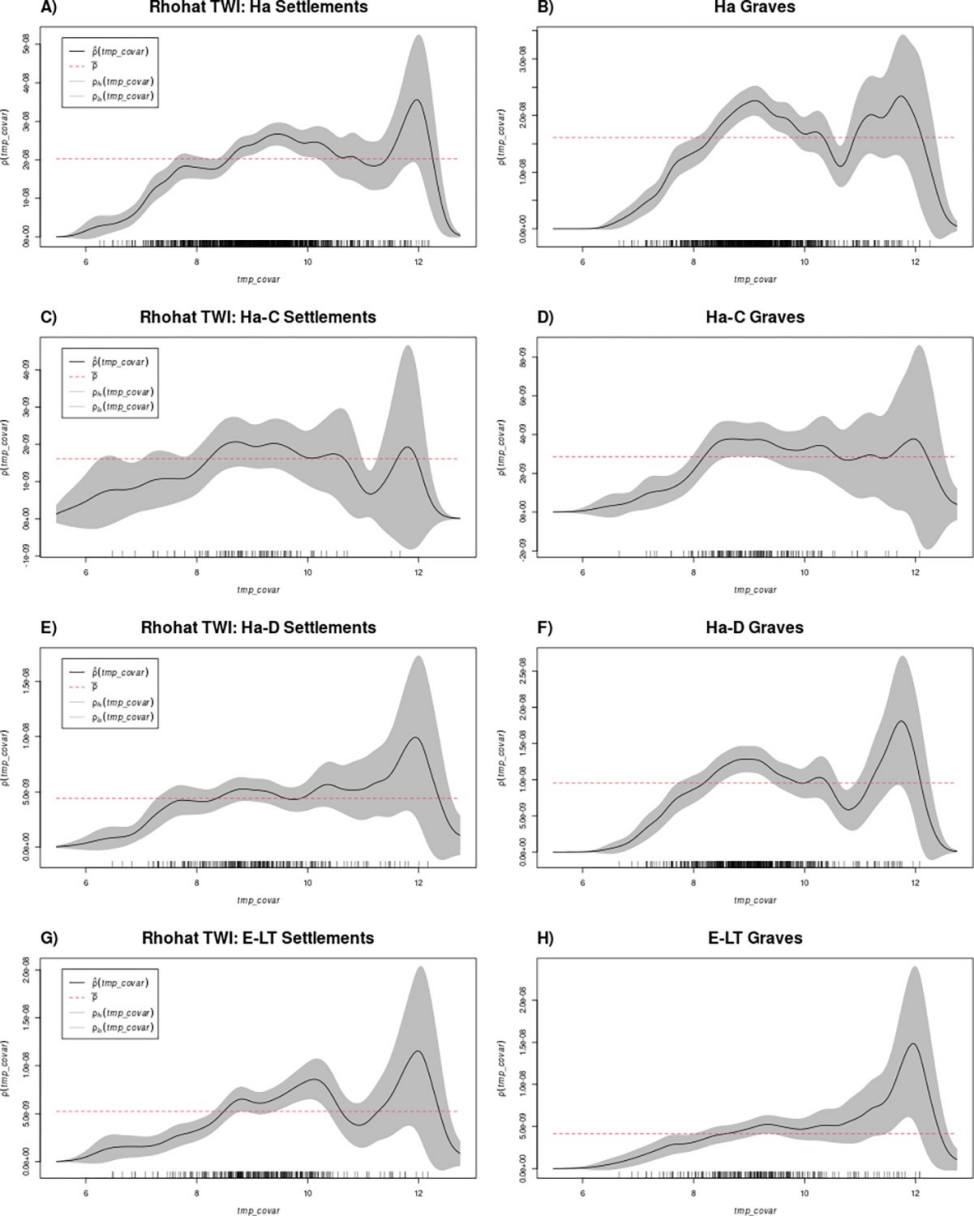
Rhohat results for settlements (left) and graves (right) vs wetness.

*Rhohat*. Hallstatt settlements are much more concentrated at elevations below approximately 400 m above sea level (asl) than expected by chance (above the red line), while they are less represented at higher elevations (below the red line, Fig 7A). This pattern holds true also when considering the periods Ha C and Ha D separately, although the intensity of sites did not differ significantly from a random distribution for higher elevations (Fig 7C and 7E). This does not match general knowledge on settlement location for Hallstatt, and more precisely Ha D, for which a higher number of hilltops compared to the previous period was assumed. Furthermore, Hallstatt settlements tend to be more common at slopes below 5°, but not around 0°. When analysed separately, sites generally fall within spatial randomness with occasional underrepresentation at higher slopes. Hallstatt settlements are found in lower than expected numbers in regions of low wetness (Fig 8A), with the bulk of sites located at intermediate values (random when phases C and D are separated, Fig 8C and 8E).

La Tène settlements exhibit similar tendencies concerning elevation and slope (Fig 7G). They were less common than expected at lower wetness but more at values around 10, indicating a preference for wetter areas, such as valleys or near rivers compared to the Hallstatt period and the random model.

When compared to distance from the rivers, settlement patterns exhibit a consistent behaviour across all historical periods. The main trend reveals that settlements tend to be located at higher intensity than expected by chance alone in proximity or at closer distance from fresh water sources (< 4000 m). These observations are similar to other studies on site distribution for different areas and time periods that also showed a preference for location in close distance to water sources or on gentle slopes [76]. Conversely, as distances increase, settlements tend to demonstrate lower intensity or remain within CSR. The only exception is represented by the Ha C, with all values below 15000 m within CSR.

The distribution of Hallstatt graves differs from that of settlements. Graves are less commonly distributed at lower elevations (<200 m asl) and intermediate elevations (400–600 m asl). Instead, a higher concentration of graves is observed at elevations of 200–400 m asl and 750–900 m asl ca (Fig 7B). This pattern persists when considering Ha C and D graves separately, with Ha C graves showing a closer-to-random distribution (Fig 7D and 7F). This result is interesting since the available literature describes a rather different site pattern, with settlements on hilltops and burials at the footslopes. This is certainly true for single case studies but not on the regional level. In this case, it is possible to suggest that their positioning reflected a desire to occupy areas with higher visibility [21,22]. The analysis of graves in relation to wetness reflects what was observed for settlements, with fewer sites at low wetness and more sites than expected at intermediate values (Fig 8B and 8F). However, in Ha C, the distribution of graves at intermediate wetness falls within the confidence interval (Fig 8D).

During the Early La Tène period, graves are found more frequently than expected at lower elevations (<350/400 m asl) and within the confidence interval at 600–1000 m asl. However, fewer sites than expected are observed at elevations between 450 and 600 m asl (Fig 7H). Compared to settlements (Figs 7G and 8G), the only remarkable difference is that at very high wetness (approximately 12) more graves than expected are recorded (Fig 8H).

**Fig 9:**
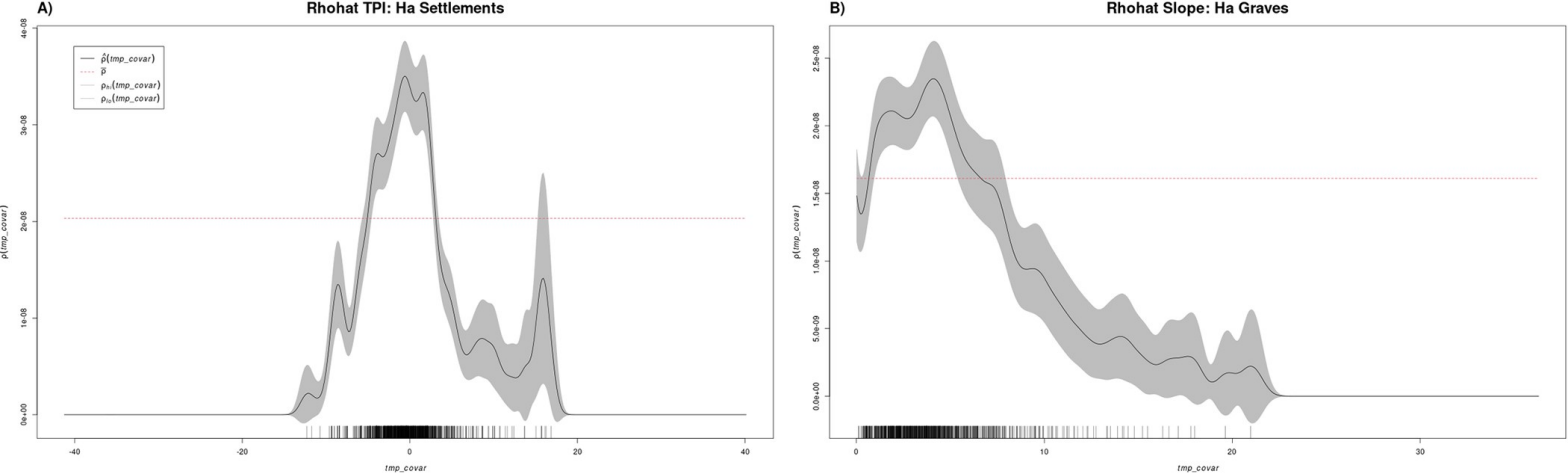
Rhohat results for Ha settlements vs TPI (left) and graves vs slope (right).

The topographic position index results show similar patterns across all periods and site types (Fig 9). Most sites are located around the value of 0, indicating no significant difference in prominence compared to the surrounding area. Conversely, fewer sites are observed for all other values of prominence (both negative and positive, see repository for the rhohat results). It is worth noting that these findings might be influenced by the radius used for prominence analysis (750 m), and further analysis considering visibility or a larger radius could yield different results (using a lower radius would instead only add noise).

The preference of occupying areas closer to rivers more intensely than expected by chance alone is observed also for graves. However, an exception is observed in the case of Ha C, where sites either align with CSR or display lower intensity at these values. In all cases, intermediate values are consistently within CSR. Finally, compared to settlements, the tendency of graves to be located at lower intensity at higher distances from rivers is similar, but only starts at values above 20000 m.

*PPM*. The results of the PPM model are summarised in Tables 1 and 2, based on the step model selecting only the most relevant covariate. Most of the covariates exhibit consistent behaviour across different periods. Slope always shows a negative correlation with site locations, aligning with our expectations. On the other hand, elevation demonstrates an inverse relationship with settlements and a positive association with graves, revealing a significant site locational pattern (Table 1). While wetness is consistently absent from the grave models, it generally displays a negative correlation with settlements, suggesting a tendency to avoid areas with higher wetness indices, such as those within river channels. Finally, when incorporated into the models, TPI consistently exhibits a negative correlation with site locations (Table 1). Archaeologically the most interesting result is that for most periods and site types, except the Ha C graves, the area with the highest predictive intensity is the river Rhine valley (Fig 6). However, only a minority of sites are located in this area. In part, this is due to the fact that many of the sites recorded in the database and located in this region are not well-dated, and therefore could not be included in the analyses. In this case, the results can be used to determine what period these sites are more likely to be dated (a ratio based on the model and the absolute number of sites known for each period). In addition, there are also two other possible explanations:

(i) The covariates we selected are not suitable and the model cannot distinguish between “suitable” or “not suitable” settlement areas; (ii) Subsequent human activity, deposition of colluvium, or riverbed relocations have destroyed or buried many archaeological sites under a thick layer of sediment. We address this in more detail in the following section on the impact of modern activity and modern biases.

**Table 1:** Results of the ppm function for settlements after performing a step function to reduce the number of covariates in each model.

Covariate	Estimate	S.E.	CI95.lo	CI95.hi	Ztest	Zval
**Halstatt Settlements**						
(Intercept)	-14.651	0.351	-15.338	-13.963	***	-41.751
dem	-0.003	0.000	-0.003	-0.003	***	-16.235
slope	-0.042	0.008	-0.057	-0.026	***	-5.154
twi	-0.114	0.033	-0.178	-0.049	***	-3.455
tpi	-0.004	0.001	-0.007	-0.002	***	-3.889
rivers	0.000	0.000	0.000	0.000	*	2.173
**Halstatt-C Setlements**						
(Intercept)	-14.389	1.262	-16.863	-11.915	***	-11.399
dem	-0.003	0.001	-0.005	-0.002	***	-5.489
slope	-0.136	0.035	-0.205	-0.067	***	-3.870
twi	-0.361	0.120	-0.596	-0.127	**	-3.015
tpi	-0.015	0.004	-0.023	-0.006	***	-3.480
**Halstatt-D Settlements**						
(Intercept)	-17.445	0.162	-17.762	-17.128	***	-107.876
dem	-0.003	0.000	-0.003	-0.002	***	-7.365
slope	-0.033	0.014	-0.061	-0.005	*	-2.293
tpi	-0.010	0.002	-0.014	-0.006	***	-4.852
**Early La Tene Settlements**						
(Intercept)	-14.571	0.697	-15.936	-13.205	***	-20.913
dem	-0.004	0.000	-0.005	-0.004	***	-11.689
slope	-0.062	0.017	-0.096	-0.028	***	-3.555
twi	-0.191	0.064	-0.317	-0.065	**	-2.973
tpi	-0.004	0.002	-0.008	0.000		-1.800

In the table, parameter estimates, standard errors, and confidence interval are shown. Negative values indicate negative correlation and the number of asterisks (*) indicates the predictor’s strength.

**Table 2:** Results of the ppm function for graves after performing a step function to reduce the number of covariates in each model.

Covariate	Estimate	S.E.	CI95.lo	CI95.hi	Ztest	Zval
**Halstatt Graves**						
(Intercept)	-17.197	0.097	-17.387	-17.007	***	-177.400
dem	0.000	0.000	0.000	0.001	*	2.110
slope	-0.112	0.010	-0.131	-0.093	***	-11.502
tpi	-0.003	0.001	-0.006	-0.001	**	-2.824
**Halstatt-C Graves**						
(Intercept)	-19.614	0.248	-20.101	-19.128	***	-79.018
dem	0.001	0.000	0.001	0.002	**	3.120
slope	-0.165	0.027	-0.218	-0.112	***	-6.076
tpi	-0.006	0.003	-0.012	0.000		-1.810
rivers	0.000	0.000	0.000	0.000	*	2.436
**Halstatt-D Graves**						
(Intercept)	-17.839	0.127	-18.088	-17.590	***	-140.632
dem	0.001	0.000	0.000	0.001	*	2.563
slope	-0.111	0.013	-0.136	-0.086	***	-8.740
tpi	-0.004	0.002	-0.007	0.000	*	-2.104
**Early La Tene Graves**						
(Intercept)	-18.016	0.123	-18.257	-17.774	***	-146.137
slope	-0.111	0.019	-0.149	-0.074	***	-5.844
tpi	-0.002	0.002	-0.007	0.003		-0.901
rivers	0.000	0.000	0.000	0.000	***	-3.429

In the table, parameter estimates, standard errors, and confidence interval are shown. Negative values indicate negative correlation and the number of asterisks (*) indicates the predictor’s strength.

Noteworthy, among the few sites located on the Rhine river is the so-called Fürstensitz of the Münsterberg in Breisach, located on a plateau of about 10 hectares and documented at least since Hallstatt C1/D. Multiple burial grounds are also known in the vicinity for Ha D and LT A, on both sides of the river Rhine [11,77]. In addition, some smaller and contemporary settlements are also known from the Kaiserstuhl area [67]. A large necropolis with several burial mounds is further identified in the north Upper Rhine Valley, but mostly located on the nowadays French side of the river and therefore excluded [68]. Most likely, these elements serve as indicators of particular site location preferences during this period correctly predicted by our model.

Furthermore, it can be observed that Ha (combined), Ha C and Early La Tène settlements are generally concentrated in the north-central part of the study area, which is correctly predicted in most of the models (see Fig 6 for Ha period and the repository for the others), with only a few sites located in the Black Forest and a relative large number of settlements along the north-western side of the river Danube in the Swabian Jura. The latter concentration can be linked to the fortified settlement of the Heuneburg and the 50 hectares of rural agglomeration and satellite dwellings that emerged, which make it one of the first proto-urban-character-like concentrations in Europe [19]. Satellite-like dwellings with agricultural cropland and multiple groups of tumuli have been suggested for the surroundings of the Heuneburg [16,78].

However, the model usually fails to predict sites in the latter region, which is geomorphologically different from the northern part of the study area with lower elevations and gentler slopes. This may be due to the heterogeneity of settlement patterns within the study area, as well as the arbitrary selection of regions (by modern administrative boundaries) with diverse characteristics that are not captured by the model. It is possible that a definition of the study area based more strictly on geomorphological characteristics would show that settlement patterns in Swabia vary consistently from the one in north-eastern Baden and central Württemberg.

The predictive model for graves does not appear to provide informative results, since in most cases a large part of the study area has relatively uniformly high values in the prediction (Table 2 and Fig 6). This result may be due to the fact that burial mounds were occupied during multiple decades or even the entire Early Iron Age [21,22]. Ha C graves may represent an exception, with most burials correctly predicted in the Swabian Jura, although the rest of the sites in the north-western part of the region are not very well predicted. This pattern may be due to the aforementioned heterogeneity of the area. However, dealing with graves may also introduce an additional layer of uncertainty due to the diverse range of sites labelled as graves in the dataset, including flat graves and burial mounds.

In general, the rhohat function and the PPM model provide a valuable basis to estimate the spatial behaviour underlying the detected point processes. However, it must be acknowledged that they are a first step towards a predictive model of site intensity based on the empirically observed point process. Hence, the spatial interaction of points and covariates can help to understand the socio-environmental links and feedbacks in archaeological and ecological research. On the other hand, the model output is strongly connected to the model character of the analytical part of the research. A model is never a real representation of certain circumstances, but rather a potential realisation of particular variables that describe a particular characterisation of an observation.

### Bias assessment

**Fig 10:**
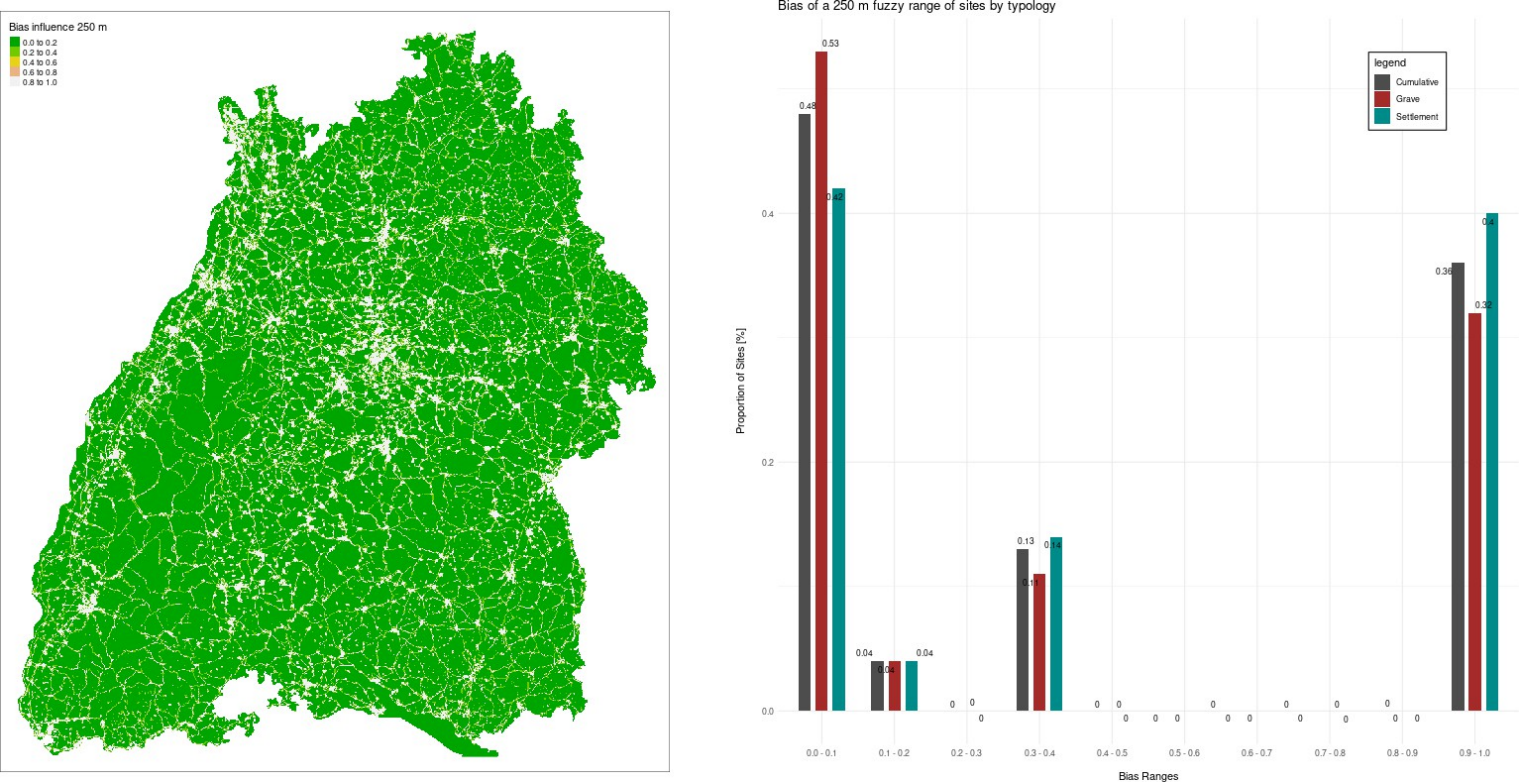
Results of the bias assessment of sites at a radius of 250 m. Data for the analyses are automatically downloaded using the script and they are provided by OpenStreetMap under the Open Database License (https://www.openstreetmap.org/copyright).

The results of the modelled bias (Fig 10) show mixed values, with approximately half of the sites located in “low bias” areas (< 0.1/0.2), while around 36% of the sites are located in areas that experienced significant modern activity. This pattern is observed for both settlements and graves and does not sensibly change when applying a 1000 or a 250 m radius to the fuzzification. However, it is important to note that settlements are the most “affected” sites, being located more frequently in areas impacted by modern activities. This is not surprising, given that settlements are less conspicuous compared to burial mounds and are often found during commercial archaeology investigations.

Additionally, it is worth mentioning that most of the biased areas are located in the central and north-western part of the study regions. Nevertheless, the results suggest that despite the presence of bias, it is still possible to study site distribution patterns, provided that archaeologists take these phenomena into account.

## Conclusions

This paper has aimed to investigate patterns in Early Iron Age site distribution from Baden-Württemberg as functions of underlying first and second order explanatory variables, and to develop prediction models for potential archaeological site distribution. In order to do so, we have applied the concept of point pattern analysis in a consistent way across the whole study region and time period. The different analyses interlock systematically and complement each other to form a picture of settlement development that contributes to a cultural-historical interpretation. While the latter is important for this key period of Central European development, we have emphasised conceptual aspects of the systematisation and reproducibility of point pattern analysis for the recognition of site patterns. It is, of course, always possible to argue for small changes or additions to make the analysis more powerful, in particular when these workflows are adopted for other case studies. Furthermore, the analyses have produced perspectives into the subjective character and biases inherent in large archaeological databases. These arise, for example, from the institutional, scientific and socio-economic priorities underlying data collection and recording. Most obviously, but not exclusively, these are highlighted in the relationship between modern land-use and recorded site dispersal. Such biases are a well appreciated feature of modern archaeology, and our model produces more finely-grained and transferable statistical and spatial insights into the phenomenon.

Despite the fact that a model may never capture the full historical complexity of site distribution and related processes, it can still provide valuable information. In the case of settlements, it is important to consider that most of these are clustered in the northern and central part of the Baden-Württemberg (see Figs 1 and 5, right), which is in general correctly predicted. The other area with a high predictive value for settlements is the Rhine valley, which, however, contains only a limited number of sites. As discussed in the results section, we know that in this area there are some very important sites (the Münsterberg at Breisach), some more are located on the French side of the river, and many others are undated, making the models more valuable. Predicting grave distribution proved to be more challenging and the results are less instructive. This can be due to the fact that cemeteries have generally a longer lifespan than settlements, and were used over generations and sometimes throughout the entire Early Iron Age. Most likely, separating burial mounds and flat graves, which surely experienced different patterns of use and site location choices, would result in more precise results, with very little increase in complexity of the model.

The analysis of spatial interaction shows many rather similar or identical results, but in particular the G-function appears to be sensitive for specific changes in site structure. This concerns the character and relationship of the clusters and the relationship of the neighbouring sites. Here, the reduction in the clustering intensity from Hallstatt C to D in the overall picture is remarkable.

The importance of rivers as main communication routes and key focal points for settlement has been extensively emphasised in the literature 64 among others] and generally confirmed in our models. This aspect is partially reflected in the locations of the density clusters and in the intensity of sites as a function of distance to rivers and wetness (Figs 6 and 9 and repository to this article for TPI).

In general, the heterogeneity of the research area of this paper presents some challenges. On one hand, using a heterogeneous area enables the examination of contrasting and different patterns. On the other hand, it can also tend to level out or smooth local patterns. A possible solution would be splitting up the area into subregions and comparing the results. This approach could also help to better account for the differences in modern activity across the region. However, it is important to note that this would result in a further decrease in the number of points in each subset, hence decreasing the statistical significance of the results. Ultimately, each case study, including the one presented in this paper, remains strongly dependent on the individual window of operation, the sample size, and the site intensity of the point process.
