# Correction: Point Pattern Analysis (PPA) as a tool for reproducible archaeological site distribution analyses and location processes in early iron age south-west Germany

**DOI:** 10.1371/journal.pone.0316867

**Published:** 2024-12-30

**Authors:** Giacomo Bilotti, Michael Kempf, Eljas Oksanen, Lizzie Scholtus, Oliver Nakoinz

The following information is missing from the Funding statement: SNSF post-doc fellow Project: EXOCHAINS—Exploring Holocene Climate Change and Human Innovations across Eurasia (SNSF grant number TMPFP2_217358.
